# The Overexpression of NMHC IIA Promoted Invasion and Metastasis of Nasopharyngeal Carcinoma Cells

**DOI:** 10.7150/jca.47506

**Published:** 2021-05-17

**Authors:** Dan Xiong, Dayang Chen, Dawei Liu, Wei Wu, Xiaowen Dou, Xiang Ji, Jian Li, Xiuming Zhang

**Affiliations:** 1 Medical Laboratory of The Third Affiliated Hospital of ShenZhen university, Shenzhen, 518001, China.; 2 Department of pathology, The First Affiliated Hospital, Sun Yat-sen University, Guangzhou, China.; 3 Department of Otolaryngology, The First Affiliated Hospital, Sun Yat-sen University, Guangzhou, China.; 4 Guangzhou Key Laboratory of Otorhinolaryngology, Guangzhou, China.

**Keywords:** NMHC IIA, head and neck squamous cell carcinoma, nasopharyngeal cancer, invasion, metastasis

## Abstract

**Background:** Nasopharyngeal carcinoma (NPC) is a kind of head and neck squamous cell carcinoma (HNSCC) with a strong tendency for metastasis and recurrence. Non-muscle myosin heavy chain IIA (NMHC IIA) plays important roles in recurrence and metastasis of cancers. However, the function and mechanism of NMHC IIA expression in NPC remain unclear.

**Methods:** A receiver operating characteristic (ROC) curve was constructed for 141 specimens of HNSCC tissues and 44 control samples from The Cancer Genome Atlas (TCGA) database. Co-expressed genes with MYH9 were identified using LinkedOmics. Transcription factors (TFs) and miRNA regulation network were constructed using Networkanalyst. The migration and invasion ability of nasopharyngeal carcinoma cells were evaluated by *in vitro* migration and matrigel invasion assays, respectively.

**Results:** The public microarray results showed that MYH9 expression levels were upregulated in HNSCC tissues compared with the matched adjacent normal tissues in this study (p<0.0001). The AUC of MYH9 reached up to 0.8303 at a cutoff value of 175.2, with a sensitivity and specificity of 70.21% and 86.36%, respectively. MYH9 expression was increased in lymph node metastasis HNSCC tumors compared with that in tumors without lymph node metastasis (p<0.05) and showed a strong positive association with expression of FLNA. High MYH9 and FLNA expression were related with poorer overall survival in HNSCC. MYH9 with positively associated genes regulated focal adhesion, cell-substrate junction assembly and cell morphogenesis were involved in differentiation using GO and KEGG analysis. MYH9 was correlated with a network of TFs including SP1, SRF, JUN and FOS in HNSCC. The suppression of endogenous NMHC IIA decreased cellular migration and invasion in HNE1 cells and reduced the expression of phosphorylation of EGFR, AKT and ERK. The over-expression of NMHC IIA increased cellular migration and invasion in COS-7 cells and increased the expression of phosphorylation of EGFR, AKT and ERK.

**Conclusion:** Expression of NMHC IIA mRNA was higher in HNSCC than in the adjacent normal tissues. NMHC IIA expression was increased in lymph node metastasis HNSCC tumors compared with tumors without lymph node metastasis. High MYH9 was association with poorer overall survival in HNSCC. NMHC IIA expression increased the invasion and metastasis abilities of the nasopharyngeal cancer cell line *in vitro* by augmenting the expression of phosphorylation of EGFR, AKT and ERK. These findings will be beneficial for providing an effectively therapeutic strategy for NPC.

## Introduction

Head and neck squamous cell carcinoma (HNSCC) contains a group of cancers arising in the oropharynx, oral cavity, nasal cavity and larynx, hypopharynx and paranasal sinuses 1. Nasopharyngeal carcinoma (NPC) is a kind of HNSCC with high incidence Epstein-Barr virus (EBV)-related epithelial malignancy, which of remarkable regional and ethnic specificity that can lead to serious health problems in south China and southeastern Asia compared with the Western world 2. According to the literature, nearly 80,000 people are diagnosed with NPC every year around the world, ranking 23^rd^ in new-onset malignant tumors 3. Approximately 50,000 people die annually of NPC 4. Most patients have reached the advanced stage at their first visit, with local lymph node and/or distant metastasis 5; therefore, early diagnosis is a major clinical problem. Currently, radiotherapy and cisplatin concurrent radiotherapy are the standard treatments for primary nasopharyngeal carcinoma 6. However, 10%-36% of patients with NPC still experience local recurrence after standard treatment 7. Patients with recurrence or metastasis after treatment often have a poor prognosis, which is the main reason for the failure of treatment and the poor survival rate of NPC patients. Therefore, screening biomarkers of NPC for early detection, the prediction of prognosis and the monitoring of recurrence are of great significance for the clinical diagnosis and treatment of NPC.

Non-muscle myosin heavy chain IIA (NMHC IIA), which is encoded by MYH9 located at chromosome 22q11.2, is a dominating subunit of the actomyosin cytoskeleton and is generally considered to contribute to contraction of the cell posterior during migration 8, 9. Many articles have reported that NMHC IIA plays an important role in tumor cell invasion and metastasis. A recent article reported that MYH9 promoted growth and metastasis in colorectal cancer 10. One study showed that the long noncoding RNA PTCSC2 binding to the MYH9 protein was more likely to lead to thyroid cancer 11. Another study showed that miR-647 inhibited the invasion and metastasis of gastric cancer cells by targeting the SRF/MYH9 axis 12. The S100A4-MYH9 axis has been reported to promote the migration and invasion of gastric cancer cells by TGF-β-mediated epithelial-mesenchymal transition 13. However, NMHC IIA has been identified as a tumor suppressor of squamous cell carcinomas 14. So, the function and mechanism of NMHC IIA in NPC development is worth studying.

In the present study, we explored the significance of NMHC IIA in NPC tumorigenesis and invasion. A receiver operating characteristic (ROC) curve was constructed by plotting the data pairs for sensitivity and (1 - specificity) using The Cancer Genome Atlas (TCGA) database. Subsequently, co-expressed genes with MYH9 were predicted by LinkedOmics 15. Gene Ontology (GO) and the Kyoto Encyclopedia of Genes and Genomes (KEGG) analysis were performed by Metascape to explore the potential regulatory functions of MYH9 16. Transcription factors (TFs) and miRNA regulation network were constructed using Networkanalyst 17. The migration and invasion abilities of nasopharyngeal carcinoma cells were evaluated by *in vitro* migration and matrigel invasion assays, respectively. Thus, our results could provide new evidence for understanding the molecular mechanisms of MYH9 in the invasion and metastasis of NPC cells.

## Materials and methods

### HNSCC patients and mRNA expression profiles

The RNA sequencing data of HNSCC and normal control samples were obtained from The Cancer Genome Atlas (TCGA, https://cancergenome.nih.gov/) database. Patient exclusion criteria were as follows: i) first histologic diagnosis was not HNSCC; ii) the presence of another malignant tumor in addition to HNSCC; iii) incomplete data for analysis; and iv) received chemotherapy or radiotherapy. Overall, 141 HNSCC patient samples and 44 normal control samples were included in this study. Table 1 shows information on the tumor stage of these tissues.

### LinkedOmics analysis

The LinkedOmics database (http://www.linkedomics.org/login.php) contains multi-omics data and clinical data for 32 cancer types. It was used to study differentially expressed genes in correlation with MYH9 in the RNAseq data type of TCGA HNSC cohort. MYH9 co-expression was analyzed statistically using Pearson's correlation coefficient, presenting in volcano plots and heat maps.

### Functional Enrichment Analysis

The Metascape (http://metascape.org) online database was used to perform a functional enrichment analysis, which included GO and the KEGG analysis. The terms with minimum count of 3, p-value < 0.01 and enrichment factor of >1.5 were set as the cutoff criterion. Protein-protein Interaction Enrichment analysis was performed using Molecular Complex Detection (MCODE) algorithm.

### Prediction of transcription factors and miRNAs

NetworkAnalyst (http://www.networkanalyst.ca) is an online visual analytics platform for comprehensive gene expression profiling. Transcription factors (TFs) and miRNAs were predicted by TF-miRNA coregulatory interactions function.

### The Kaplan-Meier Plotter Analysis

The prognostic value of the mRNA expression of MYH9 with HNSCC was evaluated using the Kaplan-Meier plotter (www.kmplot.com) 18. The overall survival of patients was determined by dividing the patient samples into two groups based on median expression (high vs. low expression). Log-rank p value < 0.05 was considered statistically significant.

### Cell culture

NPEC2 Bmi-1 cells grown in keratinocyte/serum-free (KSF) medium (Invitrogen) are immortalized nasopharyngeal epithelial cells induced by oncogene Bmi-1. All human EBV-negative NPC cell lines (SUNE1, SUNE2, 6-10B, 5-8F, CNE2, HONE1 and HNE1) and the COS-7 cell line, an African green monkey kidney fibroblast-like cell line maintained in our laboratory, were cultured in RPMI 1640 medium (GIBCO) supplemented with 5% fetal bovine serum (FBS, GIBCO). All cells were grown in a humidified 5% CO2 incubator at 37 °C and passaged using standard cell culture techniques.

### Cell transfection

Cell transfection was performed with PEI (Polysciences) according to the manufacturer's instructions. For the overexpression experiments, COS-7 cells were plated at a density of 4-6×10^4^ cells per well into 24-well plates. Sixteen hours after seeding, the cells were grown to approximately 40% confluence, and each well received 1.5 ml Fugene HD and 0.75 mg of the indicated plasmids. For the siRNA experiments, the siRNAs that targeted NMHC-IIA were obtained from Guangzhou RiboBio Co., Ltd., and a total of 2.75 × 10^5^ HNE1 cells were seeded into six-well plates. A final concentration of 50 nM siRNA duplex was reversely transfected with Lipofectamine RNAiMAX unless otherwise indicated. The sequences of the siRNAs used are as follows: NMHC-IIA siRNA #1 GCAACATCGTCTTCAAGAA and #2 ACACGGAGCTGATCAACGA.

### Knockdown Assays

The shRNAs that targeted NMHC-IIA were obtained from Sigma. Lentivirus particles were generated by transfecting HEK-293T cells with pMD2.G and psPAX2 packaging plasmids and the NMHC-IIA shRNA-encoding plasmids using PEI (Polysciences) according to the manufacturer's instructions. Viruses were collected 48 h post-transfection and filtered. A total of 2×10^5^ HNE1 cells were seeded into six-well plates. Sixteen hours after seeding, the cells were grown to approximately 40% confluence and infected with fresh lentivirus overnight in the presence of 10 µg/mL polybrene. Cells were screened by 1 µg/ml puro for 3 days. An HNE1 cell line stably silenced with NMHC-IIA was successfully established. The sequences of the short hairpin RNAs (shRNAs) used are as follows: NMHC-IIA shRNA #1 CCGGCCGCGAAGTCAGCTCCCTAAACTCGAGTTTAGGGAGCTGACTTCGCGGTTTTTG, #2 CCGGACGGAGATGGAGGACCTTATGCTCGAGCATAAGGTCCTCCATCTCCGTTTTTTG, #3 CCGGGACAGCAATCTGTACCGCATTCTCGAGAATGCGGTACAGATTGCTGTCTTTTTG and #5 CC GGGCCAAGCTCAAGAACAAGCATCTCGAGATGCTTGTTCTTGAGCTTGGCTTTTTG.

### Quantitative real-time PCR and Western blot analysis

Total RNA was isolated from cultured cells using TRIzol reagent (Invitrogen, Grand Island, NY) and reversely transcribed applying a reverse transcriptase kit (Invitrogen). The mRNA level was evaluated by real-time PCR using Power SYBR Green qPCR SuperMix-UDG (Invitrogen) and carried out using an ABI PRISM 7500 Sequence Detection System (Applied Biosystems, Foster City, CA). The procedure of the real-time PCR reaction as below: (1) 50 °C for 120 s; (2) pre-denaturation at 95 °C for 10 min; (3) denaturation at 95 °C for 15 s, annealing and extension at 60 °C for 60 s, 40 cycles. All gene expression levels were normalized to that of the housekeeping gene GAPDH as an internal standard. The forward primer for MYH9 was 5'-CCATCACAGACACCGCCTACAG-3', and the reverse primer was 5'-CTTCTTGGTGTTCTCCGTCTTGC-3'. The forward primer for GAPDH was 5'-CGAGGTCATAGTTCCTGTTGGTG-3', and the reverse primer was 5'-CCCAATACGACCAAATCCGTT-3'.

Western blot analysis was carried out as previously described 19. The Western blot was probed with polyclonal rabbit NMHC-IIA (ab13849, Abcam, 1:1,000 dilution), phospho-AKT1-Ser473 (#4060, Cell Signaling Technology, CST, 1:1,000 dilution), rabbit polyclonal anti-AKT (KC-5A01, Kangcheng Biotech., 1:1,000 dilution), p-EGFR (Tyr1068, #3777, CST, 1:1,000 dilution), EGFR (#4267, CST, 1:1,000 dilution), phospho-ERK1/2 (Thr202/Tyr204, #4376, CST, 1:1,000 dilution), p44/42 MAPK (Erk1/2) (#4695, CST, 1:1,000 dilution) and rabbit anti-GAPDH (BD, Transduction Laboratories, Lexington, UK) antibodies.

### *In vitro* matrigel invasion and migration assays

*In vitro* invasion assays were performed to measure the ability of cells to invade a matrigel matrix overlying a membrane containing an 8 µm pore size polycarbonate filter. Cells (5 × 10^4^) were seeded into medium containing 1% FBS in the top chamber coated with matrigel (BD, Transduction Laboratories, Lexington, UK), whereas medium containing 10% FBS was added to the bottom chamber. After cultivation for 48 h, the chambers were fixed with 1% paraformaldehyde and stained with hematoxylin. Invasive cells were plotted as the average number in ten random fields of view at 200× magnification for each filter. The data are expressed as the average number of cells migrating through the filters. The procedures for the migration assay were similar to those described for the matrigel invasion assay except that no matrigel was used, and the incubation time was 16 h.

### Statistical analysis

Statistical analyses were performed using the statistical software package SPSS 16.0. Differences among variables were analyzed by 2-tailed Student's t tests. Data are presented as the mean ± SD unless otherwise indicated. p values ≤0.05 were considered statistically significant.

## Results

### Expression of NMHC IIA mRNA in HNSCC and matched adjacent normal tissues from the TCGA database

**Figure 1:**
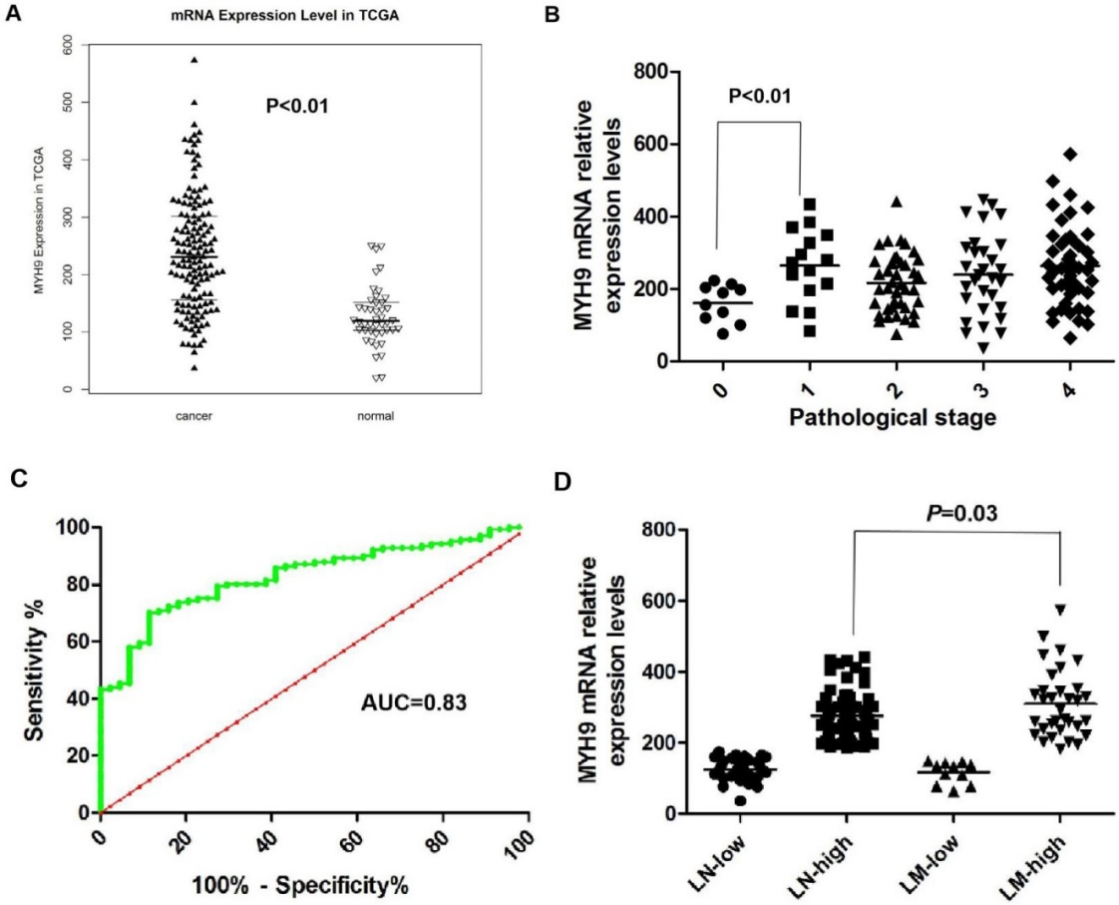
** NMHC IIA mRNA expression levels in HNSCC cancer tissues and adjacent normal tissues. A.** Enhanced expression of NMHC IIA mRNA expression levels in HNSCC cancer tissues compared with adjacent normal tissues (normal=44, cancer=141, p<0.01). **B.** NMHC IIA mRNA expression levels in HNSCC cancer of different pathological stages (T=141, p<0.01). **C.** The ROC curve for differentiating HNSCC tissues from controls. **D.** NMHC IIA had increased expression in lymph-node metastasis HNSCC compared with lymph-node non-metastasis tumors.

We previously reported that NMHC-IIA is an important factor for EBV infection in nasopharyngeal epithelial cells 20. The public mRNA microarray results showed that the NMHC IIA mRNA level was upregulated in HNSCC tissues compared with that in the matched adjacent normal tissues (p<0.01, Figure 1A). Moreover, high expression levels of NMHC IIA mRNA were observed in high clinical pathological stage (I-IV) compared with zero clinical pathological stage in HNSCC (p<0.01, Figure 1B). A ROC curve was constructed on 141 HNSCC tissue samples and 44 control specimens. The results showed that the area under the ROC curve (AUC) reached up to 0.8303 (95% confidence interval (CI)=0.770-0.891; p<0.001, Figure 1C). The optimal cutoff value was 175.2, with a sensitivity and specificity of 70.21% and 86.36%, respectively. A high level of NMHC IIA in HNSCC was significantly correlated with lymph node metastasis (p < 0.05) (Figure 1D).

### GO and KEGG pathway analyses of co-expression genes correlated with MYH9

**Figure 2:**
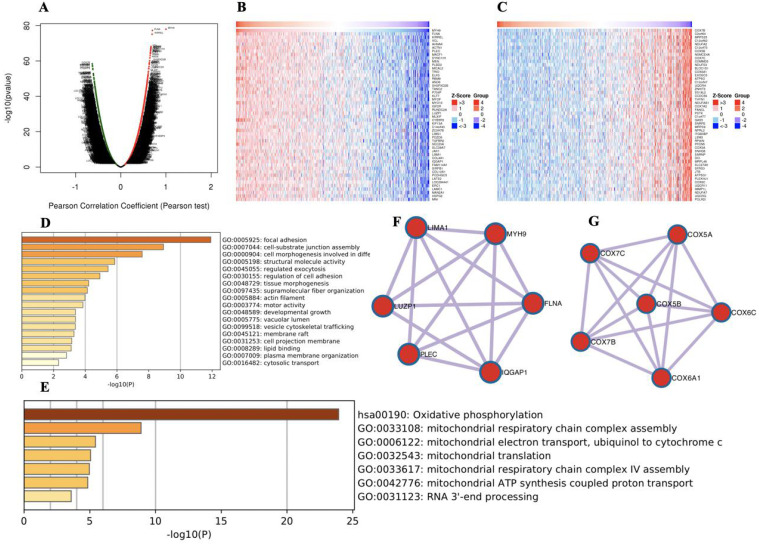
** Genes differentially expressed in correlation with MYH9 in HNSCC. A.** A Pearson test was used to analyze correlations between MYH9 and genes expressed in HNSCC. **B-C.** Heat maps showing genes positively and negatively correlated with MYH9 (TOP 50). Red indicates positively correlated genes and green indicates negatively correlated genes. **D-E.** The significantly enriched GO annotations and KEGG pathways of MYH9 of top 50 significant gene sets (D) positively and (E) negatively correlated with MYH9. **F-G.** Network module obtained from the (F) positively and (G) negatively genes protein-protein interaction network.

To further explore the functions of MYH9 in HNSCC, we analyzed the mRNA sequencing data from 520 HNSCC patients in the TCGA through LinkedOmics, as shown in the volcano plot (Figure 2A). The 50 significant genes set positively and negatively correlated with MYH9 as shown in the heat map (Figure 2B, 2C). MYH9 expression showed a strong positive association with expression of FLNA (positive rank #1, Pearson correlation = 0.70 p = 5.138e-78) and KIRREL (positive rank #2, Pearson correlation = 0.69, p = 6.868e-76). FLNA acts as a scaffold for the binding of more than 90 partners, which is critical for cell migration and adhesion^21^. GO and KEGG analysis by Metascape software showed that the top 50 significant genes set positively correlated with MYH9 were mainly enriched in focal adhesion, cell-substrate junction assembly and cell morphogenesis involved in differentiation (Figure 2D). The top 50 significant genes set negatively correlated with MYH9 were enrichment in oxidative phosphorylation, mitochondrial respiratory chain complex assembly and mitochondrial electron transport, ubiquinol to cytochrome c (Figure 2E). Subsequently, one core module was obtained from positive and negative gene PPI network using the MCODE clustering algorithm, respectively. Proteins involved in positive cluster were LIMA1, LUZP1, PLEC, IQGAP1, FLNA and MYH9 (Figure 2F). The enriched “Biological Process” GO terms contained regulation of actin filament-based process (4 genes), blood vessel development (3 genes). REACTOME pathway analysis showed that Cell-Cell communication (3 genes) was involved. Proteins involved in negative cluster were COX7C, COX7B, COX5B, COX6A1, COX6C and COX5A, which were mainly associated with mitochondrial electron transport, cytochrome c to oxygen and proton transmembrane transport in terms of Biological Process (Figure 2G).

### Transcription factors, miRNA and survival analysis of FLNA and MYH9

**Figure 3:**
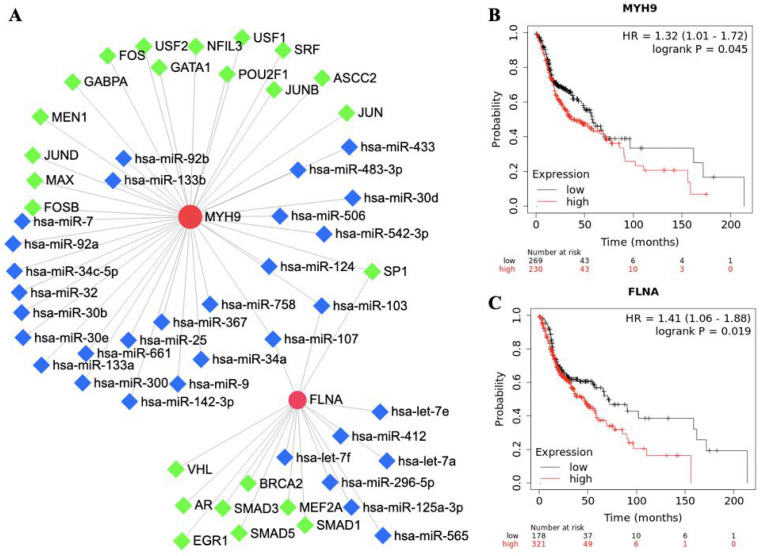
** The network and survival analysis of FLNA and MYH9. A.** Transcription factors and miRNA of FLNA and MYH9. **B-C.** Kaplan-Meier analysis of overall survival in (B) MYH9 and (C) FLNA.

The roles of FLNA and MYH9 in tumorigenesis and development have been reported in cancer. To further understand the regulatory network of FLNA and MYH9, TFs and miRNA were predicted by NetworkAnalyst. FLNA was regulated by 9 TFs and MYH9 was regulated by 15 TFs. SP1, hsa-miR-103 and hsa-miR-107 were found to regulate FLNA and MYH9 (Figure 3A). The prognostic value of FLNA and MYH9 was obtained from Kaplan-Meier plotter. High expression of MYH9 (p = 0.045, HR = 1.32) and FLNA (p = 0.019, HR = 1.41) predicted poor overall survival (Figure 3B, 3C).

### Silencing endogenous NMHC IIA reduces the migration and invasion of nasopharyngeal cancer cells

**Figure 4:**
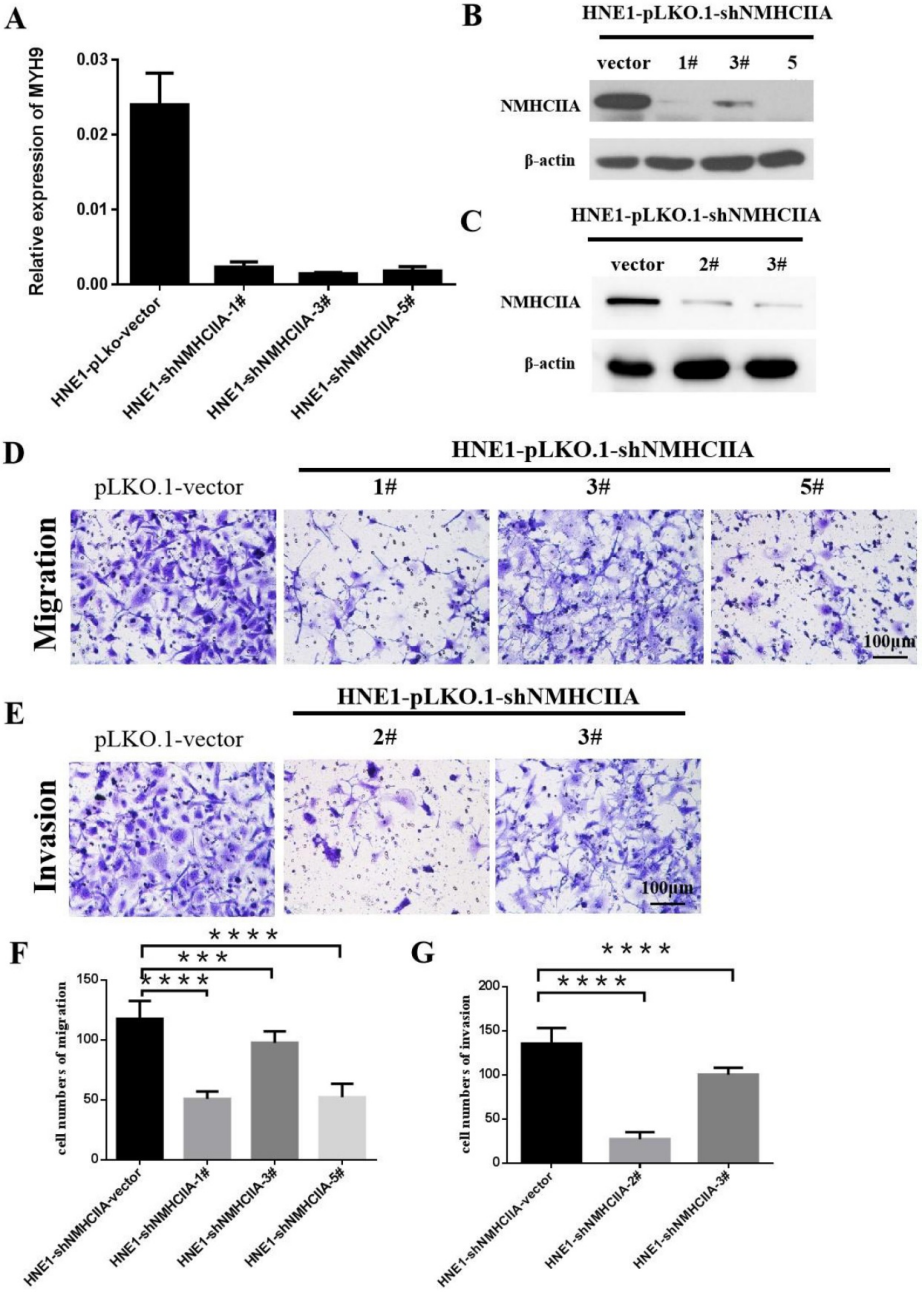
** Suppression of endogenous NMHC IIA reduced cellular migration and invasion in HNE1. A-C.** NMHC IIA expression was confirmed by Quantitative real-time PCR and western-blot in HNE1 cells expressing scrambled shRNA or NMHC IIA shRNA. **D-E.** The analysis of the migration and invasive properties in HNE1 cells expressing scrambled shRNA or NMHC IIA shRNA. The migratory or invasive cells were stained by crystal violet and then photographed by fluorescence inversion microscope system. Original magnification ×200. **F.** Migratory cells were plotted as the average number of cells per field of view from ten random fields. **G.** Invasive cells were plotted as the average number of cells per field of view from ten random fields.

Based on the above findings, we next investigated the impact of NMHC IIA on migration and invasion in nasopharyngeal cancer cells. Four short hairpin RNAs for NMHC IIA were generated to reduce NMHC IIA expression in the HNE1 cell line, as shown in Figure 4A-C. The migration assay suggested that after the knockdown of NMHC IIA, the migration capability of HNE1 cells was obviously lower than that of the control group (Figure 4D, 4F). Boyden chamber invasion assays revealed that the invasiveness of HNE1 cells was dramatically decreased by the ablation of NMHC IIA using the shRNA-mediated silencing of endogenous NMHC IIA protein (Figure 4E, 4G). Moreover, the NMHC IIA siRNA was used to decrease NMHC IIA expression in HNE1 cells, and a similar result was obtained, as shown in Supplementary Figure 1. These findings indicate that silencing endogenous NMHC IIA reduces the invasion and metastatic abilities of nasopharyngeal cancer cells.

### Overexpression of NMHC IIA enhances the migration and invasion of COS-7 cells *in vitro*

**Figure 5:**
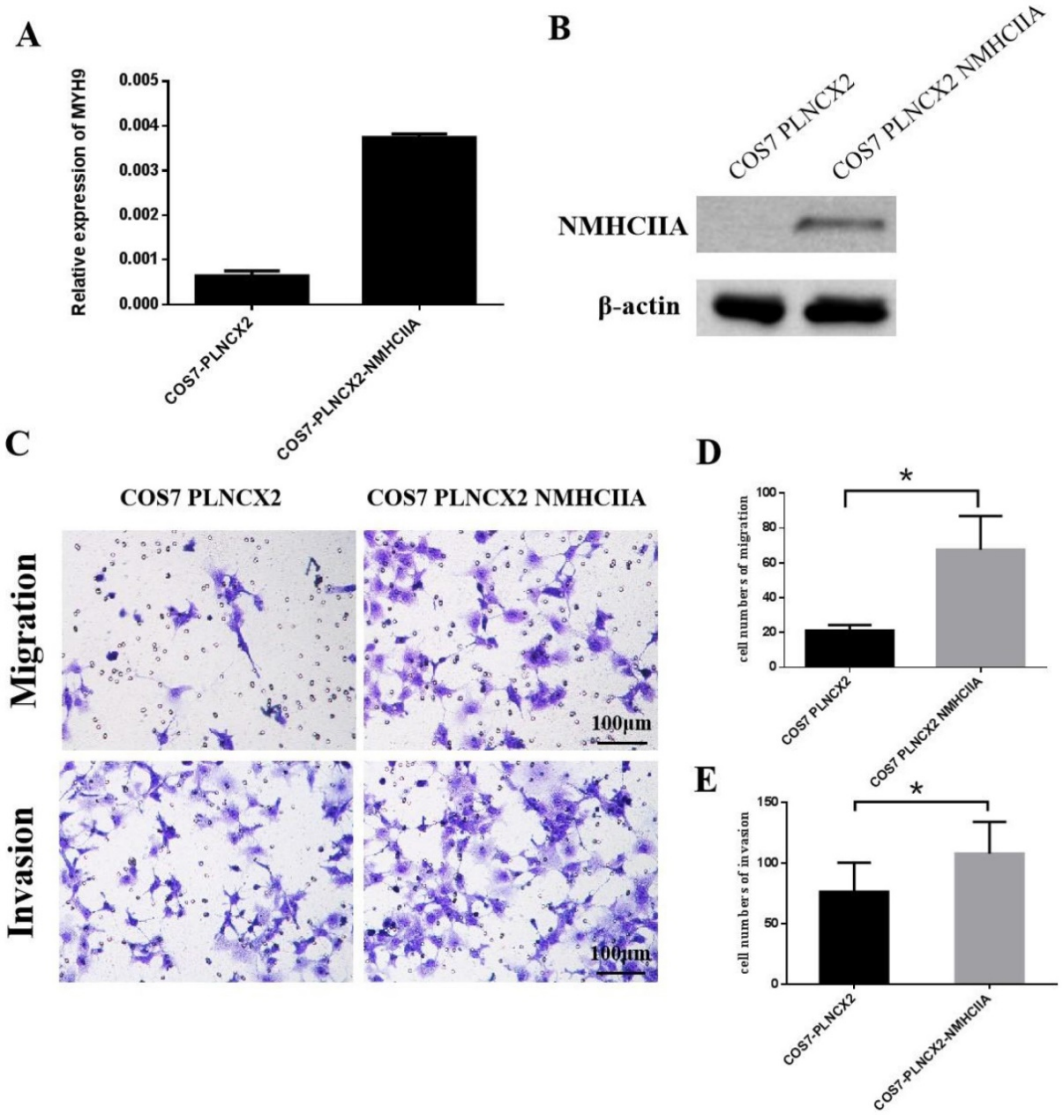
** The exogenous expression of NMHC IIA enhanced the migration and invasiveness in COS7 cells. A.** Quantitative real time PCR analysis of MYH9 gene in COS7 cells expressing control vector PLNCX2 or PLNCX2/NMHC IIA. The relative fold increase of transcripts was normalized to the amount of RNA harvested from cells expressing control vector PLNCX2. GAPDH served as the internal control. The data were presented as the mean ± SD (n = 3). **B.** Western blot analysis of NMHC IIA protein in COS7 cells expressing control vector PLNCX2 or PLNCX2/NMHC IIA. β-actin was used as a loading control. **C.** The migratory and invasive abilities induced by NMHC IIA were analyzed in COS7 cells expressing control vector PLNCX2 or PLNCX2/NMHC IIA. The migratory or invasive cells were stained by crystal violet and then photographed by fluorescence inversion microscope system (200×). **E.** Migratory cells or invasive cells were plotted as the average number of cells per field of view from ten random fields.

To further verify the influence of NMHC IIA on migration and invasion in cells, the NMHC IIA expression plasmid was stably transfected into the COS-7 cell line, which is derived from kidney tissue of the African green monkey (*Cercopithecus aethiops*) and does not express the NMHC IIA protein. Figure 5A and Figure 5B show that restored NMHC IIA expression was confirmed by real-time PCR and western blot assays in COS-7 cells. Migration and matrigel invasion chamber assays were then performed to determine the effect of NMHC IIA on the migration and invasion of COS-7 cells. The results showed that the overexpression of NMHC IIA notably increased COS-7 cell migration and invasion compared to that observed in control cells (Figure 5C-E).

### Potential mechanism induced by NMHC IIA in NPC progression

**Figure 6:**
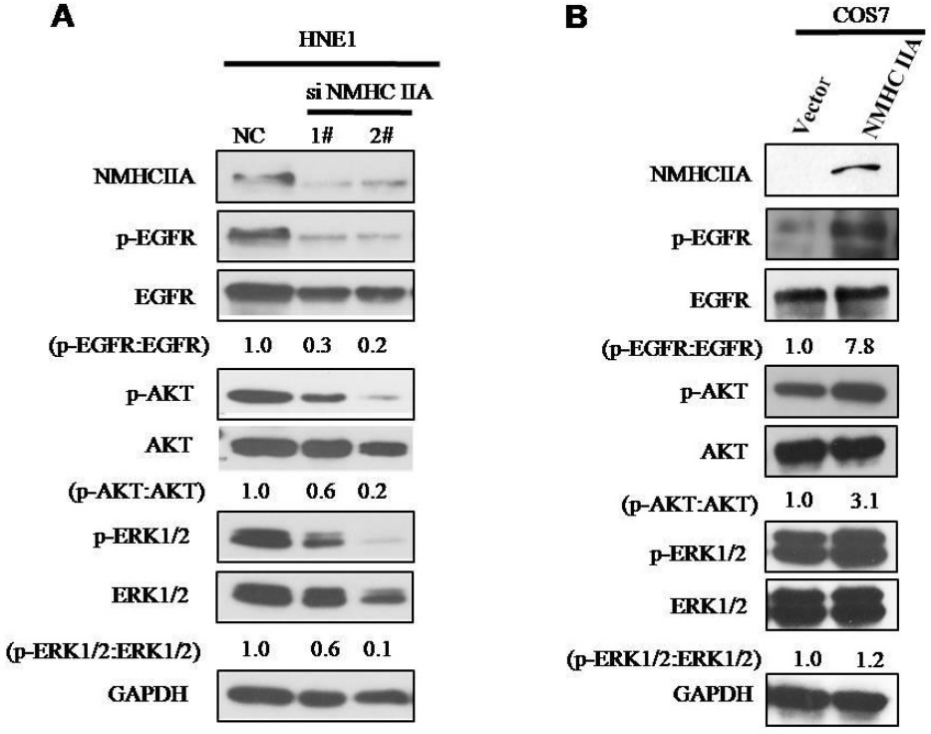
** NMHC IIA have effect on the expression of phosphorylation of EGFR, AKT and ERK. A.** Silencing endogenous NMHC IIA in HNE1 cells decreased expression of phosphorylation of EGFR, AKT and ERK. **B.** Overexpression of NMHC IIA in COS7 cells enhances the expression and phosphorylation of EGFR, AKT and ERK.

To explore the molecular mechanism underlying the effect of NMHC IIA on the migration and invasion of nasopharyngeal cells, we carried out Western blot analysis to examine the phosphorylation status of proteins associated with cancer signaling. EGFR, as well as its critical downstream signaling components AKT and ERK, is aberrantly expressed in NPC 21. As shown in Figure 6, silencing endogenous NMHC IIA in HNE1 cells decreased the expression and phosphorylation of EGFR, AKT and ERK. Overexpression of NMHC IIA in COS7 cells enhances the expression and phosphorylation of EGFR, AKT and ERK. These results suggest that increasing NMHC IIA expression could be responsible for enhancing NPC progression by inducing EGFR, AKT and ERK phosphorylation.

## Discussion

NPC is a squamous cell carcinoma that usually occurs around the osculum of the Eustachian tube in the lateral wall of the nasopharynx 22. The environmental, genetic, and viral causative factors, either acting alone or in combination, would generate multiple epigenetic and genetic alterations 23. The development of NPC involves the accumulation of multiple epigenetic and genetic changes, leading to the evolution of clonal cell populations, which have growth advantages over other cells 2. In our study, we demonstrated a significant increase in NMHC IIA expression at the mRNA level between HNSCC cancer and adjacent normal HNSCC tissues using a public data. This result was consistent with those of previous studies of other human cancers 24. Our results showed that the AUC of MYH9 reached up to 0.8303. The optimal cutoff value was 175.2, with a sensitivity and specificity of 70.21% and 86.36%, respectively. This result indicated that MYH9 may be as new candidate biomarkers for HNSCC patients by bioinformatics analysis.

To gain detailed insights into the potential functions of MYH9 in HNSCC and its regulatory network, we performed bioinformatics analysis of TCGA HNSCC RNAseq sequencing data to guide MYH9 research. We found that MYH9 expression showed a strong positive association with expression of FLNA. Filamin A (FLNa), encoded by the FLNA gene, crosslinks various signaling molecules and membrane receptors. Previous studies identified the FLNA as determinant in cancer progression and metastasis through affecting cancer cell growth and migration 25, 26, but the role of FLNA in NPC is still unknown. In our study, high expression of MYH9 and FLNA predicted poor overall survival obtained from Kaplan-Meier plotter. Moreover, FLNA and MYH9 are substrates of Mechanistic Target of Rapamycin Complex 2 (mTORC2), which is a key regulator in tumorigenesis, promoting cell growth, supporting their irregular or metastatic ability for diseased cells 27. We suspected that FLNA and MYH9 are effectors of mTORC2 controlling the motility and invasion of NPC cells.

To further explore the co-expression genes correlated with MYH9, we constructed the PPI network using top 50 significant genes positively and negatively correlated with MYH9. The positive core cluster is consisted of LIMA1, LUZP1, PLEC, IQGAP1, FLNA and MYH9. LUZP1 was identified as a FLNA binding partner by Wang et al. 28. Knockdown of LIMA1 has been demonstrated to enhance cancer cell invasion 29. IQGAP1 as a scaffold connects phosphoinositide signaling to cytoskeletal reorganization 30. Those results show that six hub genes might be essential in the function of NPC cell development. Interestingly, negative core cluster mainly regulate mitochondrial function. Cytochrome c oxidase (COX) is one of the principle enzymes in mitochondria-mediated apoptosis and cell respiration. The expression alteration of those genes may provide novel insights into the development of NPC from the perspective of energy metabolite.

We found that MYH9 in HNSCC was associated with a network of TFs including SP1, SRF, JUN and FOS. SP1 was verified as a direct functional target of miR-24, which enhances cell viability and the radiosensitivity of NPC cells 31. SRF regulates the transcriptional activity of SNAIL in NPC cells 32. Jun proteins (c-Jun, JunB, JunD) and Fos proteins (c-Fos, FosB, Fra-1, Fra-2) are belongs to Activator protein 1 (AP-1), which played an important role in the prognosis of oral squamous cell carcinoma. hsa-miR-103 and hsa-miR-107 were regulators of FLNA and MYH9. Studies have elucidated that hsa-miR-103 and hsa-miR-107 were involved in Wnt3a/β-catenin/ATF6 signaling pathway and were critical to the progression of colorectal cancer, Alzheimer's disease, breast cancer and cardiac function 33-36. The TFs and miRNA regulation network may promote understanding of the molecular mechanisms of NPC development.

More than 90% of cancer deaths are caused by metastases, not the primary tumors from which these malignant lesions arise 24, 37. Therefore, it is necessary to elucidate the potential factors of tumorigenicity, invasion and metastasis of NPC which was warranted to develop novel treatments and cures. In our study, the intense expression of NMHC IIA in HNSCC was correlated with lymph node metastasis using a bioinformatics data, while silencing endogenous NMHC IIA reduced the migration and invasion of nasopharyngeal cancer cell lines *in vitro*. This result is similar to a recent study that reported that the endogenous overexpression of NMHC IIA enhanced cell migration *in vitro* 10. Our study indicated that NMHC IIA expression was potentially responsible for enhancing NPC progression by inducing EGFR, AKT and ERK phosphorylation. Additional work is required to investigate the mechanism by which NMHC IIA regulates these pathways in the invasion and metastasis of NPC. Therefore, exploring the function and mechanism of these pathways can help us better understand the pathogenicity of NPC cells and aid in the development of more sensitive and effective diagnostic targets.

## Conclusion

In conclusion, our study revealed MYH9 as new candidate biomarkers for HNSCC patients by bioinformatics analysis. MYH9 expression was strong positive association with expression of FLNA using TCGA HNSCC RNAseq sequencing data. High MYH9 and FLNA expression were associated with the poorer overall survival in HNSCC.MYH9 was positively correlated genes regulate focal adhesion, cell-substrate junction assembly and cell morphogenesis involved in differentiation by performing GO and KEGG analysis. MYH9 was associated with a network of TFs including SP1, SRF, JUN and FOS in HNSCC. Moreover, silencing endogenous NMHC IIA reduced the migration and invasion of the nasopharyngeal cancer cell line *in vitro* by inhibiting the phosphorylation of EGFR, AKT and ERK, while overexpression of NMHC IIA notably increased cells migration and invasion by enhancing the phosphorylation of EGFR, AKT and ERK. These results indicated that the transcript levels of MYH9 may be a suitable biomarker of NPC.

## Supplementary Material

Supplementary figure S1.

## Figures and Tables

**Table 1:** The pathological stage information of HNSCC cancer tissues and adjacent normal tissues from TCGA database in this study.

HNSCC tissues	
Stage	Number
NA	10
Stage I	15
Stage II	40
Stage III	30
Stage IVa	43
Stage IVb	2
Stage IVc	1

## Abbreviations

HNSCC: Head and neck squamous cell carcinoma
NPC: Nasopharyngeal carcinoma
NMHC IIA: Non-muscle myosin heavy chain IIA
ROC: A receiver operating characteristic
TFs: Transcription factors
PPI: protein-protein interaction network
