# Supplementary figures and images for: The Overexpression of NMHC IIA Promoted Invasion and Metastasis of Nasopharyngeal Carcinoma Cells
Source: J Cancer. 2021 May 17;12(14):4218–28. doi: 10.7150/jca.47506 (PMC8176418; doi:10.7150/jca.47506)

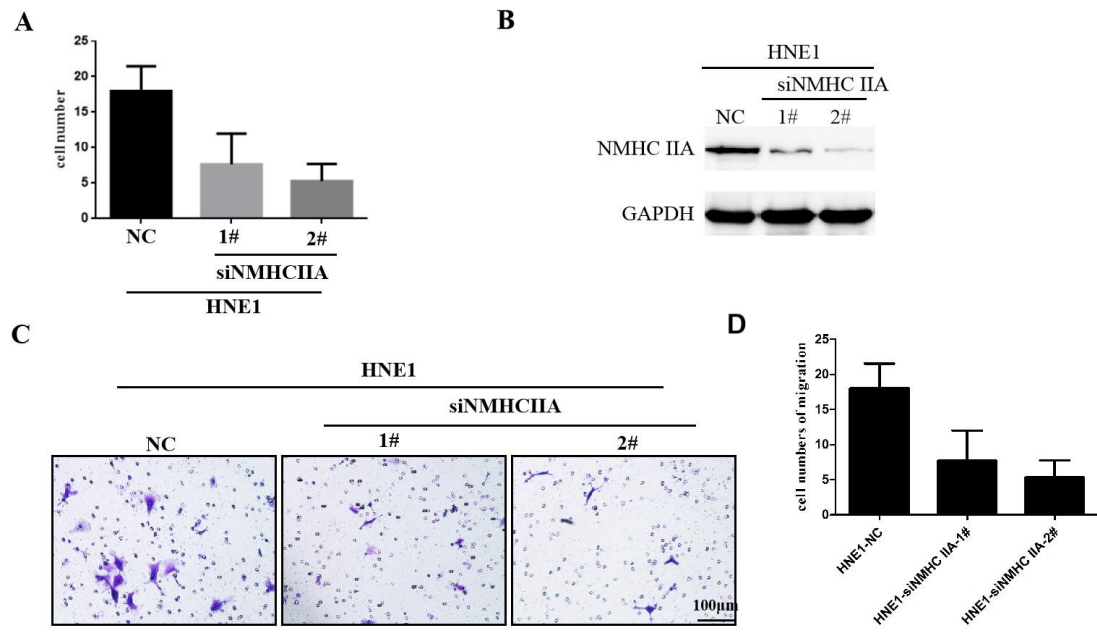

Supplement: Supplementary file 1 — Supplementary figure S1. [file jcav12p4218s1.pdf]
